# Giant photon bunching, superradiant pulse emission and excitation trapping in quantum-dot nanolasers

**DOI:** 10.1038/ncomms11540

**Published:** 2016-05-10

**Authors:** Frank Jahnke, Christopher Gies, Marc Aßmann, Manfred Bayer, H. A. M. Leymann, Alexander Foerster, Jan Wiersig, Christian Schneider, Martin Kamp, Sven Höfling

**Affiliations:** 1 Institute for Theoretical Physics, University of Bremen, 28334 Bremen, Germany; 2 Experimentelle Physik II, Technische Universität Dortmund, 44221 Dortmund, Germany; 3 Institute for Theoretical Physics, Otto-von-Guericke University of Magdeburg, 39016 Magdeburg, Germany; 4 Technische Physik, University of Würzburg, 97074 Würzburg, Germany; 5 School of Physics and Astronomy, University of St Andrews, KY16 9SS St Andrews, UK

## Abstract

Light is often characterized only by its classical properties, like intensity or coherence. When looking at its quantum properties, described by photon correlations, new information about the state of the matter generating the radiation can be revealed. In particular the difference between independent and entangled emitters, which is at the heart of quantum mechanics, can be made visible in the photon statistics of the emitted light. The well-studied phenomenon of superradiance occurs when quantum–mechanical correlations between the emitters are present. Notwithstanding, superradiance was previously demonstrated only in terms of classical light properties. Here, we provide the missing link between quantum correlations of the active material and photon correlations in the emitted radiation. We use the superradiance of quantum dots in a cavity-quantum electrodynamics laser to show a direct connection between superradiant pulse emission and distinctive changes in the photon correlation function. This directly demonstrates the importance of quantum–mechanical correlations and their transfer between carriers and photons in novel optoelectronic devices.

## Abstract (web summary)

Classical light sources are characterized by their intensity and coherence, whereas quantum light sources are described by photon correlations. Here, the authors provide a connection between the two for the case of superradiant emission from quantum dots in a nanolaser.

## Graphical abstract

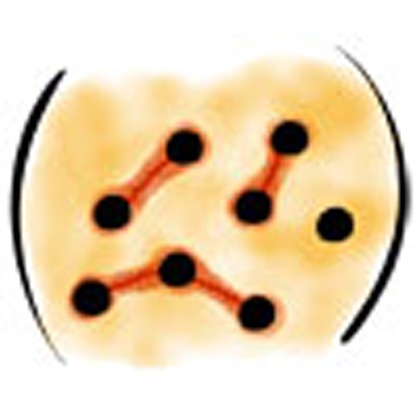

Thermal radiation is found in the uncorrelated spontaneous recombination of independent emitters. Quantum mechanically, this type of light can be distinguished from coherent (above threshold) laser emission or the more exotic nonclassical light states using the second-order photon correlation function, 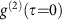. The latter is two for thermal light, one for coherent emission, and zero for a single-photon source. Despite being widely known^1^, the experimental demonstration of second-order photon correlations changing from two to one in the threshold transition of a laser was only recently possible in semiconductor nanolasers^2^ due to the required high-time resolution in connection with the fast decay of photon correlations.

Investigating the connection between the emission process and photon correlations is even more appealing for superradiance. The latter results from a collective emission process, on the basis of a correlated state of the active material, which is spontaneously established via exchange of photons between the emitters. Despite being an extensively studied phenomenon^3^,^4^ observed in a variety of systems, including semiconductor quantum dots^5^,^6^, practically all demonstrations of superradiance so far rely on macroscopic properties: changes of the time-resolved intensity or linewidth of the emitted radiation. Most prominent is the transition of the time dynamics from the exponential decay of independent emitters to superradiant pulse emission as a result of collective-emitter decay^3^, although most experiments resort to decay-time changes as function of the emitter number. The recent interest in superradiance of superconducting qubits^7^, trapped atoms^8^ and semiconductor magneto-plasmas^9^ was driven by the possibility to study directly the correlated state of the active material. In ref. 7, the limit of two emitters has been realized with superconducting qubits. Their entanglement was shown to be the origin for superradiant emission. For an ensemble of entangled emitters, the connection between superradiance and photon correlations has been analyzed in ref. 10. In case of many trapped atoms, their correlated state was used to demonstrate superradiant laser action with ultranarrow linewidth^8^. By influencing the self-organization of dipoles in a semiconductor magneto-plasma^9^, superradiant pulse emission was enabled or destroyed.

In this work we demonstrate that radiative emitter coupling can dramatically change the statistical properties of the light emission. These changes provide a more direct way to study the quantum–mechanical inter-emitter correlations driving the superradiance and the underlying physics of the collective decay. In ref. 11, for quantum-dot emitters coupled to a single-mode optical cavity, it has been predicted that a signature of the joint multi-emitter eigenstates established by radiative coupling is enhanced photon bunching (super-thermal emission) in the cavity radiation. The effect diminishes with increasing number of emitters, and is strongly hampered by electronic dephasing processes^12^. For the trapped-atom system coupled to a single optical mode, the theory in ref. 13 corroborates enhanced photon bunching and predicts emission with ultranarrow linewidth usable for ultrastable clock applications. The latter has been confirmed subsequently in ref. 8, while experimental demonstrations of the enhanced photon bunching have been missing so far.

Our system represents the solid-state analogue to trapped atoms in an optical or microwave cavity. We use semiconductor quantum-dot emitters, which act as artificial atoms with discrete emission lines. The emitters are embedded in an optical resonator. The system is designed as a monolithic laser device with a miniaturization driven to the level where the spatial dimensions of the resonator approach the light wavelength. In this strongly reduced mode volume, only a small number of emitters is resonantly coupled to the optical mode. As such, our investigations carry recent studies on fundamental quantum–optical systems over to highly miniaturized nanolaser devices. Semiconductor cavity-quantum electrodynamics (QED) lasers have been used to demonstrate coherent emission with strongly reduced laser threshold^14^,^15^ and lasing in the strong coupling regime^16^. So far, cavity-QED laser properties have not been related to superradiance of the active material.

The central finding is that our system does not behave like a conventional laser. We have identified three independent signatures of dominating inter-emitter coupling, which determine the emission properties below threshold and in the threshold region for pulsed optical pump excitation: superradiant pulse emission with a temporal duration more than one order of magnitude faster than the spontaneous lifetime of individual emitters, giant photon bunching in the second-order photon correlation function 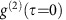 strongly exceeding the value of two for thermal light, and excitation trapping suppressing the emission by almost two orders of magnitude as long as the correlations between the emitters are present. All three signatures are simultaneously confirmed in the experiment.

## Results

### Nanolaser design and experimental findings

The used nanolaser consists of two distributed Bragg reflectors defining the photon confinement in vertical (emission) direction. In the cavity field antinode a single sheet of quantum dots is placed. With the fabrication of pillars, an efficient optical mode confinement in horizontal directions is provided by total internal reflection. We use pillars with 6 μm diameter, Q-factor of 5,250, and about 200 quantum-dot emitters within a 1 meV window of the cavity resonance. The recently explored non-resonant emitter-cavity coupling effect^17^,^18^ provides efficient contributions from slightly detuned emitters. For far-off-resonant optical excitation of the quantum dots, picosecond pulses from a Ti-sapphire laser set to 780 nm at a repetition frequency of 75.39 MHz are used. The excitation pulses are focused onto a single pillar resonator using a microscope objective with a numerical aperture of 0.26. The same objective is used for collecting the micropillar emission. At the sample position, the excitation spot has a diameter of ∼10 μm and covers the whole pillar. The ground mode emission is selected by an interference filter with 1-nm bandwidth. Further details regarding the sample, experimental conditions, and photon correlation measurements are provided in the Methods section.

**Figure 1:**
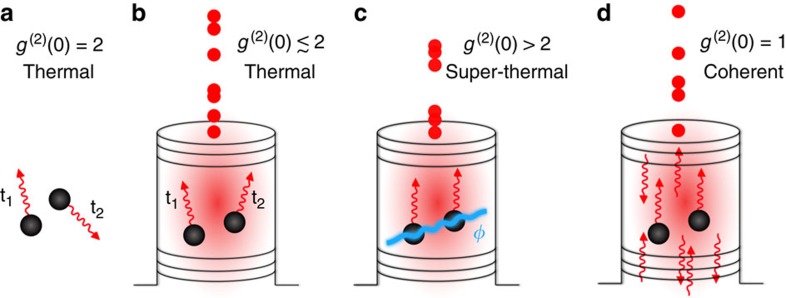
Illustration of emission regimes. (**a**) Spontaneous recombination from independent emitters leads to thermal radiation. (**b**) Using three-dimensional photon confinement in a cavity-quantum electrodynamics laser, spontaneous emission is directed into a single resonator mode. For independent emitters, below threshold the photon emission is uncorrelated, producing thermal or close to thermal light. (**c**) The exchange of photons introduces correlations between the electronic states of different emitters. A relative phase information *ϕ* is spontaneously established, and the emission from this entangled many-particle state leads to a superradiant pulse with giant photon bunching. (**d**) Above threshold, stimulated emission dominates and leads to coherent radiation.

**Figure 2:**
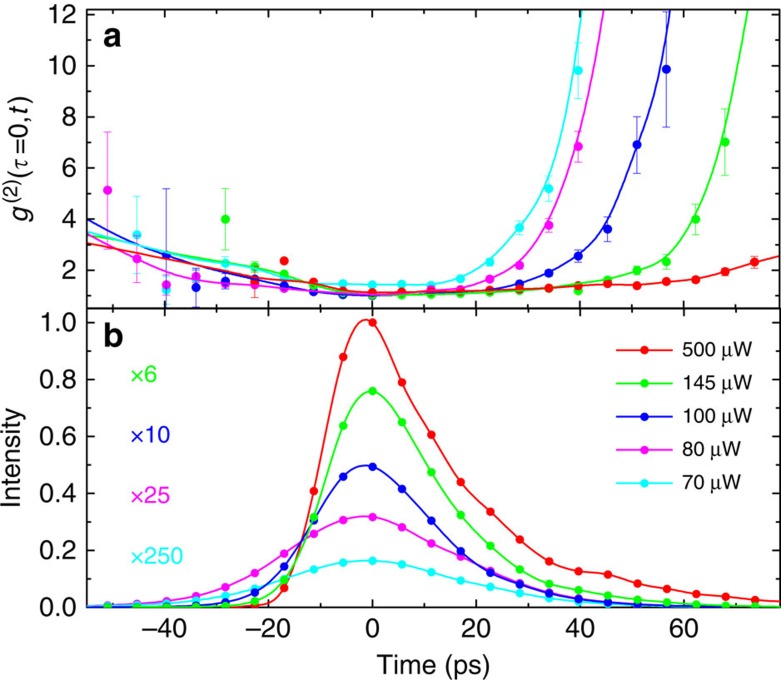
Experimental results for different emission regimes in a micropillar laser. Measurements of the intensity autocorrelation function 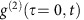 (**a**) and time-resolved output intensity (**b**) after pulsed excitation of the nanolaser with various intensities. For each pump intensity, zero time is shifted to the output-pulse maximum. The lines are guides to the eye. Error bars denote the standard counting error.

The goal of our experiment is to distinguish different emission regimes sketched in Fig. 1. For this purpose, a short excitation pulse creates carriers in energetically higher states of the active material. These excited carriers are rapidly captured into the quantum dots within few picoseconds^19^. The subsequent carrier recombination from the discrete quantum-dot states leads to the observed emission pulse, shown in Fig. 2. Surprisingly, even for the weakest applied pump power (70 μW), the emission pulse is considerably shorter (on the order of 20 ps) than the spontaneous recombination time of the individual quantum dots. The latter is about 200 ps in the cavity when taking the Purcell effect into account. To identify the nature of this short emission pulse, the time evolution of the photon correlation function 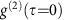 can be used. During the emission-pulse maximum, 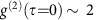 identifies thermal radiation, while before and in particular after the peak, 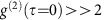 is obtained. Clearly this pulse is not linked to stimulated emission and through a direct comparison with the theory discussed below, it can be identified as the superradiant pulse. For 80 μW pump power, stimulated emission is reached during the emission-pulse maximum, as 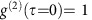 is found there. However, right before and after the peak, emission changes to thermal radiation and most of the pulse wings carry the signatures of superradiance with enhanced photon bunching. Only for the highest pump rate (500 μW), during the whole emission pulse 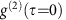 remains close to 1, identifying the output as a coherent laser pulse. Clearly the pulse shape and duration are only slightly changing with increasing pump and output intensity. However, photon correlation measurements allow us to identify different emission regimes.

### Microscopic theory

**Figure 3:**
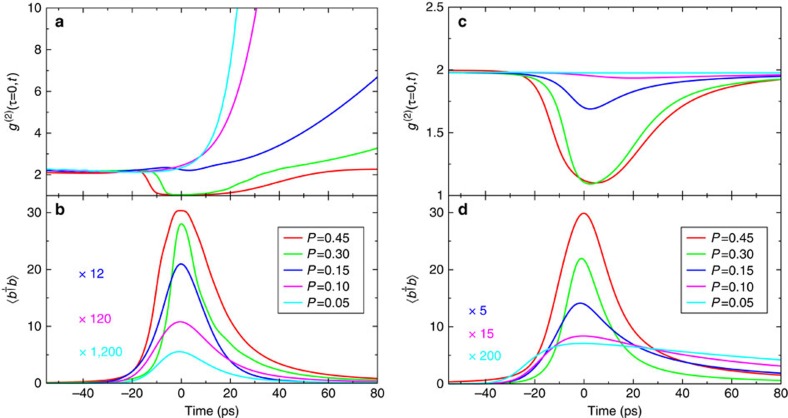
Comparison of theoretical results with and without superradiant coupling. Time evolution of the intensity autocorrelation function 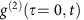 (**a**,**c**) and mean photon number (**b**,**d**) after pulsed excitation of a nanolaser with various pump-pulse areas *P*. Different curves correspond to below-threshold excitation (*P*=0.05), the transition region (*P*=0.1 and 0.15), and above-threshold excitation (*P*=0.3 and 0.45). In **a**,**b** the superradiant coupling between different quantum dots is included, while in **c**,**d** it is omitted.

To analyze the connection between electronic correlations among different emitters and quantum–mechanical photon correlations, we use results of a microscopic theory. It includes a non-perturbative description of the Jaynes–Cummings interaction between emitters and cavity field in the presence of various dissipation processes due to cavity losses, carrier recombination with emission into other modes, as well as pump and carrier scattering processes. For the treatment of cooperative emission from 200 emitters, a numerical solution of the von-Neumann–Lindblad equation of the density matrix for electronic and photonic excitations (Master equation), as used in refs 11, 12, is not feasible due to the huge state space. Our theoretical results are on the basis of a coupled equations of motion hierarchy for expectation values of carrier-, photon- and carrier–photon correlation functions, which can be derived using the cluster expansion technique^20^,^21^,^22^,^23^. A simplified version of this theory has been used in refs 24, 25 to calculate 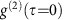 for quantum-dot nanolasers. Present extensions used here include the configuration-picture description in terms of multi-exciton states, a systematic treatment of various dephasing processes via Lindblad terms^21^, and the inclusion of inter-emitter correlations responsible for superradiance. To model the electronic states of the quantum dots, we use a four-level scheme shown in Fig. 1 of ref. 26, which also contains the connection of the levels by various interaction processes. Further details of the theory are provided in the Methods section. Results are presented in Fig. 3 for material parameters corresponding to the experimental situation (see Methods).

For weak pumping, *P*=0.05, the key observations are thermal radiation during the emission-pulse maximum and the development of giant photon bunching in association with the considerable output-pulse shortening, as seen in the experiment. In the theory, superradiant coupling can be switched off by omitting all correlation functions describing the entanglement of different emitters. In this case (right part of Fig. 3), the emission remains thermal during the whole time evolution with 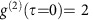 and the time-resolved intensity exhibits an exponential decay with the much longer spontaneous-emission lifetime of individual emitters. For increasing pump-pulse area *P* the system reaches stimulated emission during the output-pulse maximum. When stimulated emission starts to dominate the system dynamics, the superradiant coupling is destroyed.

**Figure 4:**
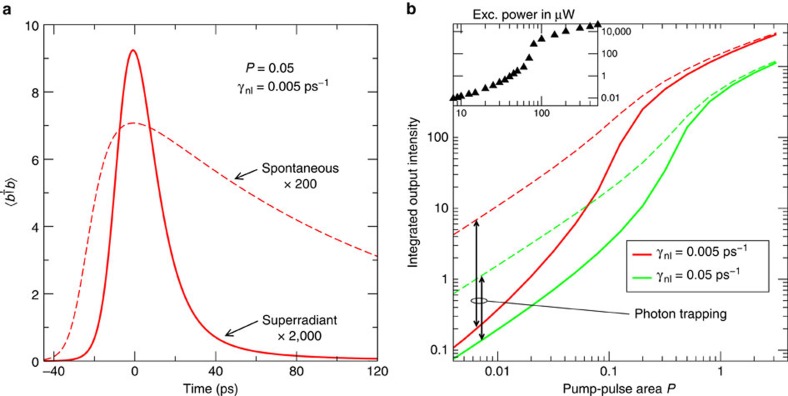
Superradiant pulse emission and excitation trapping. Calculated time evolution of the cavity mean photon number after pulsed optical excitation for weak pumping (**a**) and time integrated output intensity versus pump-pulse area (**b**). Results including superradiant coupling of the emitters (solid lines) are compared to those for independent emitters (dashed lines). As indicated in **b** a stronger spontaneous-emission rate into non-lasing modes *γ*_nl_ decreases the threshold modification due to superradiant coupling but corroborates the robustness of the effect. The inset confirms the presence of the threshold-kink in the experiment despite a large *β*-factor as an indication of excitation trapping due to superradiant emitter coupling.

A direct comparison of the calculated emission pulse with and without cooperative effects is presented in the left part of Fig. 4 for weak pumping. The radiation of independent emitters into the cavity mode follows the much slower spontaneous lifetime, while the cooperative dynamics lead to a faster de-excitation of the system in the form of a superradiant emission pulse. We also find that the superradiant coupling strongly reduces the mean photon number in the cavity mode as long as the radiative emitter coupling is prevailing, that is, below threshold and in the broadened threshold region (right part of Fig. 4). In this case, the excitation is stored as collective coherence among the emitters rather than in the cavity field. In a simple picture, photons are exchanged among emitters, thereby driving an inter-emitter polarization, and thus are not present in the cavity mode. With stronger spontaneous emission into non-lasing modes *γ*_nl_=0.05 ps^−1^, the threshold shifts to higher pump rates and the excitation trapping is reduced.

## Discussion

A simple rate equation analysis^27^, on the basis of uncoupled and non-saturating emitters, predicts that the threshold-kink in a double-logarithmic plot of the input–output power curve scales with 1/*β*. Here *β* is the ratio of spontaneous emission into the laser mode over the total spontaneous emission. This behaviour is often used to estimate the *β*-factor in nanolasers. Our results demonstrate that superradiant coupling of emitters obscures this property. The experiment is on the basis of a high *β*-laser with a spontaneous-emission coupling efficiency around 0.1 due to strong three-dimensional photon confinement. Nevertheless, the experimental input–output power curve in the inset to the right part of Fig. 4 shows a jump over more than two orders of magnitude. Such a pronounced jump is also obtained from the theory including superradiant emitter coupling (red solid curve, right panel of Fig. 4), while for a calculation assuming independent emitters (red dashed curve) the kink is almost absent. The difference between both curves indicates that superradiant emitter coupling is most strongly developed in the spontaneous-emission regime and persists during the gradual transition into stimulated emission, while the effect is destroyed once stimulated emission dominates. Our results underscore the importance of radiative inter-emitter coupling for macroscopic nanolaser device properties and their quantitative modelling.

**Figure 5:**
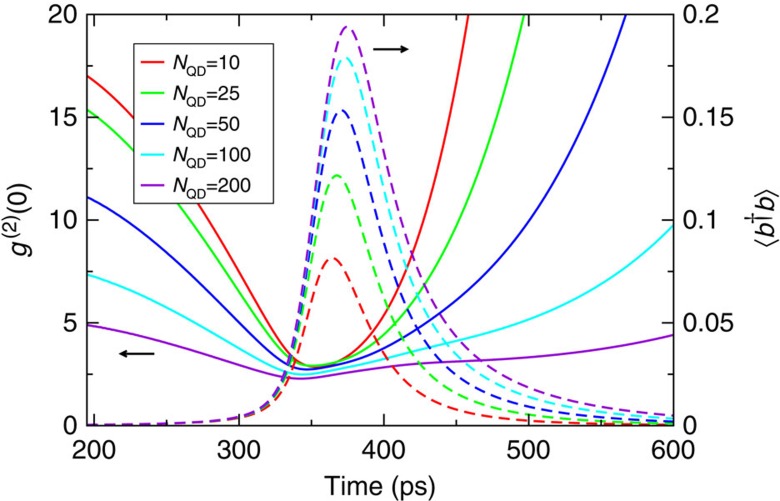
Theoretical results for different emitter numbers. Time evolution of the mean photon number 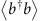 and intensity autocorrelation function 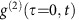 for a pump-pulse area *P*=0.1, spontaneous recombination rate in other modes *γ*_nl_=0.01 ps^−1^, and quantum-dot number *N*_QD_.

In conventional superradiance, where the emitters collectively radiate into free space, the superradiant pulse duration has a characteristic inverse proportionality to the emitter number^3^, which is not found in the presence of the cavity, see Fig. 5. The strength of the superradiant pulse increases with emitter number, while the photon bunching is stronger for a smaller emitter number. In the limit of two emitters, the joint eigenstates provide equal probability for exciting a bright state and a dark state. Through further excitation, the latter leads to the emission of two subsequent photons as the origin of the observed photon bunching^11^. With the joint states of more emitters, the probability of photon pairs decreases. While with fewer emitters, the quantum correlations due to radiative emitter coupling are stronger, these correlations create a faster emission rate, but also trap some of the photons. These counter-acting effects lead to a delay of the emission pulse almost (but not exactly) independent of the quantum-dot number.

In previous studies, the effect of radiative coupling between the emitters has been demonstrated by changing the emitter number. This is difficult and error-prone for semiconductor quantum dots, as changing the area density in the growth process usually affects other properties like size, confinement energies, dipole-couplings and dephasing, thereby influencing the superradiant coupling. Modifying the emitter number by changing the micropillar laser diameter affects cavity parameters like *β* and *Q*. A clear advantage of the present study is the availability of the three independent signatures for the radiative coupling introduced above. We have confirmed all three of them by demonstrating (i) a superfast spontaneous-emission pulse with emission-rate enhancement by a factor of 10, (ii) giant photon bunching and (iii) excitation trapping leading to large kinks in the input–output curve, which are two orders of magnitude larger than expected from the system *β*-factor. In the microscopic theory, all three signatures are explained by radiative inter-emitter coupling and disappear when this coupling is omitted.

In conclusion, this work shows how the quantum-correlated state of an emitter ensemble translates during the process of superradiance into correlations of the emitted photons. The time-resolved second-order photon correlation function is used to identify various emission regimes of the active material below, at, and above lasing threshold. It should be possible to extend these investigations to other systems in which superradiance so far has been identified on macroscopic/classical properties of the emitted radiation. In view of the specifically studied system of cavity-QED nanolasers, our results shed new light on the physics underlying the device operation. In these highly miniaturized lasers with small mode volume, a small number of atom-like emitters, strongly reduced laser threshold and low intra-cavity photon number, inter-emitter correlations directly influence typical laser properties by increasing the threshold-kink or shortening the emission-pulse duration of the dynamical response close to threshold.

## Methods

### Experiment and sample

The experimental determination of photon correlations directly relies on sufficiently high-time resolution, as 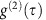 decays for thermal light to unity as a function of 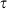 (the time between different single-photon counting events) on the scale of the coherence time. For thermal light the latter is about 10 ps. We use a streak camera in single-photon counting mode to obtain a track record of the individual photons. By introducing time bins of 2 ps size, which define the time resolution, the temporal evolution of the probability for two-photon events is determined. Photon correlation functions are calculated from averaging over ten thousands of cycles. Details about the raw data evaluation and comparable examples for the statistical quality of the data can be found in refs 28, 29. Furthermore, it has been verified that the results for 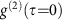 are not influenced by external noise, shot to shot fluctuations of the excitation laser or similar effects. The influence of external noise on 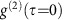 has been studied in ref. 30. In general, we find that external noise tends to increase 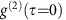 at the position of the strongest nonlinearity due to the dynamics becoming slightly faster or slower depending on the pump power within each shot. Accordingly, an additional enhancement of 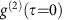 would occur on the rising edge of the pulse, where the gradient of the average intensity is steep, while only a small increase in 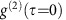 will occur in the tail, where the intensity gradient is moderate. This is actually exactly opposite to what we observe in experiment, where 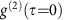 shows the strongest increase in the tail, where superradiance is expected.

For the experiment the sample was inserted in a helium-flow cryostat and cooled to a temperature of 10 K. The studied microresonators consist of upper and lower Bragg mirrors with 20 and 23 alternating *λ*/4 pairs of AlAs (79 nm) and GaAs (67 nm), respectively. The *λ* cavity between the Bragg mirrors contains a single layer of self-assembled InGaAs/GaAs quantum dots with a density of ≈3 × 10^10^ cm^−2^. Due to composition and size variations, the quantum dots exhibit a natural fluctuation of transition energies. For emission into free space, which involves a quasi-continuum of optical modes, inhomogeneous broadening of emitters rapidly diminishes the effect of superradiance due to destructive interference. In this paper, we study radiative emitter coupling mediated by a high-Q cavity mode. In this case, inhomogeneous broadening is less detrimental. A peculiarity of the used quantum-dot emitters is the recently explored non-resonant coupling effect in which detuned quantum dots can still couple into the cavity mode under the participation of longitudinal acoustic phonons^17^ or by using the energy of additional carriers in the quantum dots^18^. Hence, it is the combination of inter-quantum-dot coupling mediated by the cavity mode and the possibility of the detuned quantum dots to emit photons into the cavity mode (rather than at the detuned quantum-dot transition energy) that facilitates efficient radiative quantum-dot coupling. In our case, the radiative emitter coupling is present for weak and intermediate pumping levels, that is, in the regime of below-threshold spontaneous emission. In this regime, the quantum-dot population is weak and the non-resonant coupling effect due to additional carriers in the quantum dots with a larger detuning range^18^ should be of minor importance. Our estimate for the number of contributing quantum dots is based on non-resonant coupling due to phonons^17^, which has a smaller detuning range of about 1 meV. It should be noted that only the small fraction of quantum dots subject to the non-resonant coupling effect forms the set of active emitters, that participate in the cavity-mode light-matter interaction, and only their emission into non-lasing modes contributes to *γ*_nl_.

From the above considerations the number of quantum dots involved in the cavity emission can be estimated as follows: The total number of dot structures in the cavity is about 8,500, which is obtained from the dot density and the cavity area. However, only a fraction of these structures contributes to the luminescence monitored through a cavity mode. First, the inhomogeneous broadening of the emission amounts to 21 meV, compared to the spectral window of 1 meV considered for the non-resonant dot-cavity coupling for the relevant low pumping levels. Second, the cavity mode is located on the high-energy flank of the inhomogeneous dot distribution, where the emission intensity has dropped to about half of the emission intensity in the maximum. Correcting for these factors gives an estimate of 200 quantum dots involved in the cavity-mode emission. Possible small variations of the actual dot number may explain some deviations of the calculations from experiment, cf. Fig. 5 where we investigate the effect of the emitter number systematically. Nevertheless, the basic results of the enhanced bunching, the accelerated emission and the remarkable jump in the input–output curve remain unaffected.

The consistency of our experimental results for the three independent superradiance criteria was confirmed on several micropillar lasers of different diameter, which were processed out of the same waver.

### Additional experiment results

**Figure 6:**
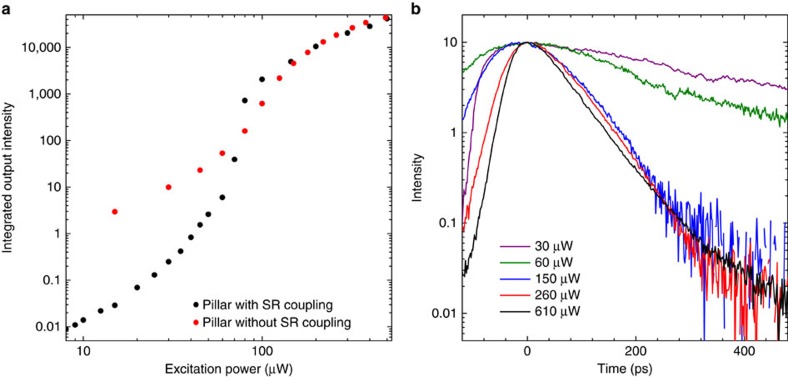
Experimental comparison of nanolasers with and without superradiant coupling. Measured output intensity versus pump power for pulsed excitation (**a**). The black symbols correspond to the data shown in Fig. 2 and represent a nanolaser with superradiant coupling between its InGaAs quantum-dot emitters. The red symbols are taken from a measurement on another sample with similar cavity parameters (comparable cavity-Q-factor and pillar diameter) but different active material based on AlInGaAs quantum dots. Here, the emitters do not exhibit superradiant coupling, as indicated in (**b**) showing the time-resolved emission for various excitation powers.

With the super-thermal photon bunching and superradiant pulse emission, we have already presented two independent signatures for the presence of radiative emitter coupling. In order to verify the influence of radiative coupling on the input–output power curve, we compare in Fig. 6a experimental results from two different samples with and without the coupling effect. In both cases, similar optical resonators have been used, but with different quantum-dot emitters. The black symbols represent the sample for which the time-resolved emission after pulsed optical excitation is shown in the bottom part of Fig. 2, and which exhibits the signatures of radiative emitter coupling as discussed in the main text. In this sample, the active material is based on InGaAs quantum dots with an In-content of 30% and a diameter of 20–30 nm, resulting in a ground-state emission energy of 1.38 eV. These quantum dots show strong carrier confinement, as evidenced by a sublevel splitting of about 40 meV. In the other sample, the emitters are replaced by quaternary AlInGaAs quantum dots, having a larger band gap. In order to obtain the same optical transition energies, enlarged dots were fabricated in the form of islands with an in-plane asymmetry having a long (short) axis of about 80 nm (40 nm) size. These quasi-two-dimensional dot structures show a similar ground-state emission energy but the carrier quantization is much weaker with a sublevel splitting on the order of 5 meV. As a result, the confined excitons are also much more susceptible to dephasing, which in turn should suppress the radiative coupling between the emitters.

The red symbols in Fig. 6a are obtained from this sample, for which the time-resolved emission is shown in Fig. 6b. In this case and contrary to the previous result, weak excitation leads to a slow and approximately exponential decay with a decay time of 450 ps, about a factor of 20 longer than for the radiatively coupled quantum dots. For elevated pump powers, the time evolution transforms into a short laser pulse. When different pillar diameters are fabricated for this type of sample (that is, with the same active material), the exponential decay time for weak excitation changes with the mode volume according to the Purcell effect. Hence, for this type of cavity one can identify clear signatures of the emission from independent quantum dots. The absence of radiative emitter coupling in this second type of sample can have several origins. Enhanced compositional quantum-dot fluctuations, as expected for the transition from ternary to quaternary material, can lead to variations of energy levels and dipole coupling strengths. In our case, the two types of quantum-dot ensembles show similar inhomogeneous broadening to which, however, multiple factors contribute. For example, possible composition fluctuations may be counterbalanced by reduced sensitivity to dot size fluctuations. Further, modified electronic confinement and the corresponding level structure change can lead to an enhanced dephasing rate. We tentatively assign the suppressed radiative coupling to this enhanced dephasing in the structure with the AlInGaAs dots.

When comparing the double-logarithmic plot of the input–output power curve between the samples with and without superradiant emitter coupling, the threshold-kink is enhanced by about two orders of magnitude in agreement with the theoretical prediction in Fig. 4b.

### Theory

Our microscopic model is based on a configuration-picture description of the quantum-dot multi-exciton-states in order to account for various optically bright and dark excitations. The emitters have a small number of discrete states for electrons and holes, which can be populated according to the Pauli principle. Typical optically bright electronic configurations include excitons and biexcitons, which can appear in their ground and excited states. The state vector 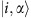 represents electronic configurations *i* in the emitter α. Expectation values of the projection operators 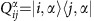 describe for *i*=*j* the population of the configuration *i* and for *i*≠*j* transitions from the configurations *j* to *i*. Most prominent among the 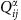 are exciton creation (annihilation) operators 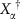 (*X*_*α*_) representing transitions between the empty quantum dot *α* and the lowest electron–hole-pair excitation. Photons in the cavity mode are described in terms of creation and annihilation operators, *b*^†^ and *b*, respectively.

Examples for expectation values contributing in the theory are 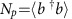 (mean cavity photon number), 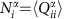 (occupation probability of electronic configuration *i*) and 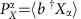 (photon-assisted transition amplitude, representing photon generation due to exciton recombination). The second-order photon correlation function is calculated from 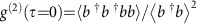. Radiative coupling between different emitters is established by processes in which the exciton recombination in emitter *β* leads to the exciton generation in emitter *α* via the exchange of a cavity photon. The result of these processes is a spontaneous build-up of a coherent amplitude between the quantum dots. The fundamental correlation function describing the superradiant coupling is 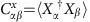. It plays the role of a generalized order parameter and reflects a macroscopic quantum coherence established between different emitters. Note that 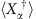 remains zero for the considered incoherent excitation of the quantum-dot states, while 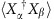 builds up as a result of spontaneous emission and reabsorption events. In the equations of motion this is reflected via driving terms linked to 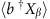. Furthermore, 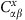 is coupled to other expectation values like 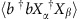, which represents the inter-emitter coherence in the presence of cavity photons and 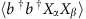 describing two-photon emission events, which are related to the photon bunching.

For our calculations we consider a set of identical quantum dots containing two single-particle levels (ground and excited states) for electrons and holes (four confined states in total). We use the same electronic configurations and dissipative processes discussed in detail for the single emitter case in ref. 26 except that here we exploit equations of motions that approximate the time evolution of the density matrix for the much larger system of *N*_QD_ quantum dots and cavity photons. On quadruplet level, the full hierarchy for all possible electronic and photonic correlation functions consists of 287 coupled equations, which have been determined and coded using computer algebra.

For the numerical evaluation of the theory, we use material parameters according to the experiment. The cavity decay rate for a Q-factor of 5,250 is *κ*=0.4 ps^−1^, corresponding to a photon lifetime of about 2.5 ps. The quantum-dot radiative lifetime as measured on a pure quantum-dot sample is 450 ps. Inside the resonator this lifetime becomes shortened to 200 ps due to the Purcell effect. This corresponds to a spontaneous recombination rate *γ*_nl_=0.005 ps^−1^. A coupling rate *g*=0.1 ps^−1^ for the quantum dots to the high-Q cavity mode (laser mode) has been used.

Four-wave-mixing studies on similar samples show a homogeneous linewidth of 3–4 μeV, which arises mostly from radiative decay. Due to the lattice distortion the resulting zero-phonon line is accompanied by phonon sidebands, which are important for the off-resonant cavity-mode feeding. Intraband carrier relaxation with the rate *γ*_rel_ in connection with the generation of excited electrons and holes in the quantum dots additionally contributes to the homogeneous broadening.

In order to model optical pumping of the quantum-dot barrier states and subsequent carrier relaxation in the experiment, a lorentzian pump pulse with a full width at half maximum (FWHM) of 20 ps is used^26^. The carrier relaxation rate itself increases with the excitation of the system as studied in detail in ref. 19. Since the microscopic treatment of interaction-induced dephasing is a subject in its own and beyond the scope of this work, we consider here a simple functional dependence between the carrier relaxation rate 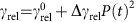 and the time-resolved pump rate *P*(*t*). Our experimental situation is well described for Δ*γ*_rel_=10 ps^−1^ and 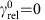 except for the largest pump pulse area *P*=0.45, where 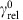
=2 ps^−1^ yields better agreement. This choice reflects the presence of carriers in delocalized states for larger pump rates, which act as additional scattering partners.

### Glossary

Spontaneous emission of independent emitters leads to thermal radiation. The corresponding photon statistics yields a mean photon number following a Bose–Einstein function, and the associated population decay of the emitter is exponential with a time constant defined as the spontaneous lifetime. Stimulated emission requires the presence of additional photons; it takes place for independent emitters and is even possible with a single emitter. Its main characteristic is the resulting coherent state of the radiation. In 1954, R. Dicke discovered the phenomenon of superradiance in which the collective spontaneous emission results in a modified radiative lifetime and the possible emergence of a superradiant emission pulse^31^. The origin of superradiance lies in the quantum–mechanical eigenstates of the joint system, the so-called Dicke states. They possess strong quantum entanglement^10^, which is the origin of inter-emitter correlations. These correlations are established by the exchange of photons between the emitters. An example for inter-emitter correlations is the expectation values for a process in which one emitter performs a ground-to-excited-state transition, whereas another emitter is de-excited in the opposite way. Such an inter-emitter polarization forms a generalized order parameter and describes a spontaneously established quantum coherence in the system.

## Additional information

**How to cite this article:** Jahnke, F. *et al*. Giant photon bunching, superradiant pulse emission, and excitation trapping in quantum-dot nanolasers. *Nat. Commun.* 7:11540 doi: 10.1038/ncomms11540 (2016).
